# Role of NLRP3 Inflammasomes for Rhabdomyolysis-induced Acute Kidney Injury

**DOI:** 10.1038/srep10901

**Published:** 2015-06-05

**Authors:** Takanori Komada, Fumitake Usui, Akira Kawashima, Hiroaki Kimura, Tadayoshi Karasawa, Yoshiyuki Inoue, Motoi Kobayashi, Yoshiko Mizushina, Tadashi Kasahara, Shun’ichiro Taniguchi, Shigeaki Muto, Daisuke Nagata, Masafumi Takahashi

**Affiliations:** 1 Division of Inflammation Research, Center for Molecular Medicine; 2 Department of Nephrology, Jichi Medical University, Tochigi; 3 Department of Molecular Oncology, Shinshu University Graduate School of Medicine, Nagano; Japan

## Abstract

Rhabdomyolysis is one of the main causes of community-acquired acute kidney injury (AKI). Although inflammation is involved in the pathogenesis of rhabdomyolysis-induced AKI (RIAKI), little is known about the mechanism that triggers inflammation during RIAKI. Recent evidence has indicated that sterile inflammation triggered by tissue injury can be mediated through multiprotein complexes called the inflammasomes. Therefore, we investigated the role of NLRP3 inflammasomes in the pathogenesis of RIAKI using a glycerol-induced murine rhabdomyolysis model. Inflammasome-related molecules were upregulated in the kidney of RIAKI. Renal tubular injury and dysfunction preceded leukocyte infiltration into the kidney during the early phase of RIAKI, and they were markedly attenuated in mice deficient in NLRP3, ASC, caspase-1, and interleukin (IL)-1β compared with those in wild-type mice. No difference in leukocyte infiltration was observed between wild-type and NLRP3-deficient mice. Furthermore, NLRP3 deficiency strikingly suppressed the expression of renal injury markers and inflammatory cytokines and apoptosis of renal tubular cells. These results demonstrated that NLRP3 inflammasomes contribute to inflammation, apoptosis, and tissue injury during the early phase of RIAKI and provide new insights into the mechanism underlying the pathogenesis of RIAKI.

Acute kidney injury (AKI) is a common problem worldwide. Recent clinical evidence has suggested that even a minor increase in serum creatinine (Cr) can be associated with poor patient survival^1^,^2^ and that severe or repeat AKI may transition to chronic kidney disease (CKD) and end-stage renal disease (ESRD)^3^. Rhabdomyolysis is one of the important causes of community-acquired AKI and accounts for 5%–15% of AKI, and rhabdomyolysis-induced AKI (RIAKI) is a systemic syndrome caused by muscle damage and the subsequent release of intramuscular contents into the peripheral circulation^1^,^2^,^4^. For decades, the mechanisms of RIAKI have been explained by direct or indirect toxicity of iron-binding heme protein, myoglobin. Myoglobin released from the injured muscle is deposited in renal proximal tubular cells and induces necrosis as a consequence of the ferrous iron release and oxidant injury. Intratubular obstruction with myoglobin casts also participates in the renal tubular injury. In addition, the scavenging of nitric oxide by myoglobin can induce renal vasoconstriction and aggravate renal hypoperfusion. As another mechanism, recent investigations indicate that inflammation plays a key role in the pathogenesis of RIAKI^5^,^6^. However, little is known about the mechanisms that trigger inflammatory responses during RIAKI.

Increasing evidence also indicates that inflammatory responses in the absence of pathogens, known as sterile inflammation, are mediated through the inflammasomes; these are intracellular multi-protein complexes that regulate proinflammatory cytokine interleukin (IL)-1β release^7^,^8^,^9^. Although many types of inflammasomes have been identified to date, the NLRP3 inflammasomes are the best characterized and have been implicated in sterile inflammation^10^,^11^. The NLRP3 inflammasomes contain NLRP3 associated with an apoptosis-associated speck-like protein containing a caspase recruitment domain (ASC), which recruits caspase-1 and induces its activation. Since caspase-1 is known as an IL-1β-converting enzyme (ICE), it processes pro-IL-1β into its mature form, which is then released, causing inflammatory responses and tissue damage. Our group recently demonstrated the importance of NLRP3 inflammasomes in the sterile inflammatory responses of vascular injury^12^, atherosclerosis^13^, abdominal aortic aneurysm^14^, myocardial ischemia-reperfusion (I/R) injury^15^, and nanoparticle-triggered pregnancy complications^16^. Our findings have been further supported by recent works showing that NLRP3 inflammasomes are key mediators of other sterile inflammatory diseases including gout, pseudogout, asbestosis, silicosis, Alzheimer’s disease, metabolic syndrome, and type 2 diabetes (see review^10^,^11^). With respect to kidney diseases, we have recently demonstrated that ASC deficiency attenuated renal inflammatory responses after unilateral ureteral obstruction and identified that collecting duct epithelial cells were responsible for inflammasome activation in the kidney^17^, suggesting a critical role for NLRP3 inflammasomes in inflammatory responses that can cause renal disease progression. Therefore, we hypothesized that NLRP3 inflammasomes can mediate inflammatory responses in the process of RIAKI. To test this hypothesis, we used mice deficient in NLRP3 inflammasome-related molecules, such as NLRP3, ASC, caspase-1, and IL-1β, and generated a glycerol-induced RIAKI, which can be an excellent model for human RIAKI. The findings obtained from this study showed the importance of NLRP3 inflammasomes in RIAKI and provide new insights into the mechanisms underlying the pathogenesis of RIAKI.

## Results

### Expression of NLRP3 inflammasome-related molecules in RIAKI

**Figure 1:**
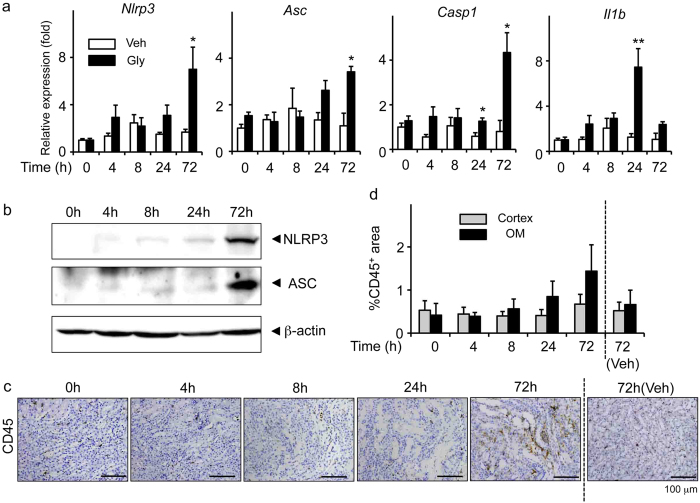
Renal expression of NLRP3 inflammasome-related molecules in RIAKI. Mice were sacrificed at the indicated time points after 5 mL/kg glycerol (Gly) or vehicle (Veh) administration. Total RNA and protein were prepared from the kidneys of WT mice. (**a**) Renal mRNA levels of *Nlrp3*, *Asc*, *Casp1*, and *Il1b* were assessed by real-time RT-PCR analysis (n = 3–5 for each). (**b**) Renal expression of NLRP3 and ASC was assessed by Western blot analysis. β-actin was used as the loading control. (**c** and **d**) Leukocyte infiltration was assessed by immunostaining for a pan-leukocyte marker CD45. Representative photographs are shown (**c**). Quantitative analysis of CD45-positive area in the cortex and outer medulla (OM) was performed (n = 3–5) (**d**). Data are expressed as mean ± SEM and analyzed using unpaired *t* test vs. vehicles. *p < 0.05, **p < 0.01.

We first determined if NLRP3 inflammasome-related molecules were upregulated in the kidney during RIAKI. Real-time reverse transcription-polymerase chain reaction (RT-PCR) analysis revealed that the renal expression of *Nlrp3*, *Asc*, and *Casp1* mRNA was significantly increased at 72 h after the induction of RIAKI, whereas the expression of *Il1b* tended to be upregulated in a time-dependent manner, reached a peak at 24 h (*p* < 0.01), and declined thereafter (Fig. 1a).We also detected that the NLRP3 and ASC protein levels were slightly detected during 4–24 h and clearly increased at 72 h (Fig. 1b), whereas no changes of NLRP3 expression in the vehicle-treated kidneys was observed until 72 h (Supplementary figure 1). Because NLRP3 inflammasomes are mainly activated in infiltrated leukocytes^8^,^18^, we next examined leukocyte infiltration in the kidney by immunohistochemical analysis using antibodies against CD45 and found that the infiltration of CD45-positive leukocytes was prominent at 72 h (Fig. 1c,d). These results suggest that the upregulation of NLRP3 inflammasomes at a later phase of RIAKI seems to be accompanied by an increased infiltration of leukocytes in the kidney.

### Renal injury in RIAKI

**Figure 2:**
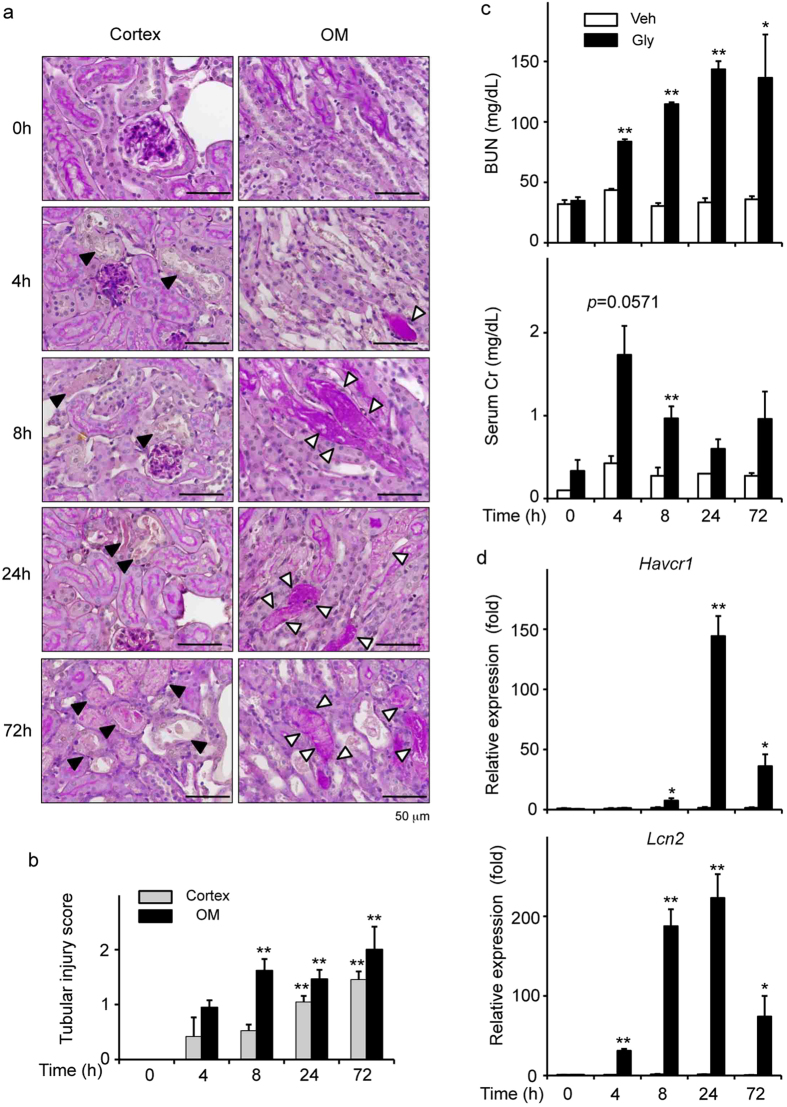
Renal injury in RIAKI. Mice were sacrificed at the indicated time points after 5 mL/kg glycerol (Gly) or vehicle (Veh) administration. (**a**) Representative images of PAS staining in the cortex and outer medulla (OM) are shown (black arrowheads, lytic lesions of the tubular epithelium; white arrowheads, intratubular casts). (**b**) The tubular injury score in the cortex and OM was determined (n = 3 for each). (**c**) Serum levels of BUN and Cr were assessed (n = 3–5 for each). (**d**) Renal mRNA levels of *Havcr1* and *Lcn2* were assessed by real-time RT-PCR analysis (n = 3–5 for each). Data are expressed as mean ± SEM. *p < 0.05, **p < 0.01.

To time-dependently analyze the structural damages in RIAKI, periodic acid-Schiff (PAS) staining was performed. Tubular injury including necrotic lysis, loss of brush border, and tubular casts were visualized from 4 h after the induction of RIAKI (Fig. 2a,b). The serum level of blood urea nitrogen (BUN) was also increased in a time-dependent manner (Fig. 2c). Because creatinine (Cr) is released from the damaged muscle during RIAKI^19^, serum Cr levels were increased at 4 h. The renal expression of *Havcr1* and *Lcn2*, which encode AKI biomarkers kidney injury molecule-1 (KIM-1) and neutrophil gelatinase-associated lipocalin (Ngal)^20^ respectively, was markedly upregulated at 8 or 24 h after RIAKI induction (Fig. 2d). There results suggest that renal tubular injury and dysfunction precede inflammatory cell infiltration during RIAKI. To explore how NLRP3 inflammasomes may be involved at the early phase of RIAKI, the following experiments were conducted at 24 h after RIAKI induction.

### Effects of deficiency of NLRP3 inflammasome-related molecules on renal injury and leukocyte infiltration

**Figure 3:**
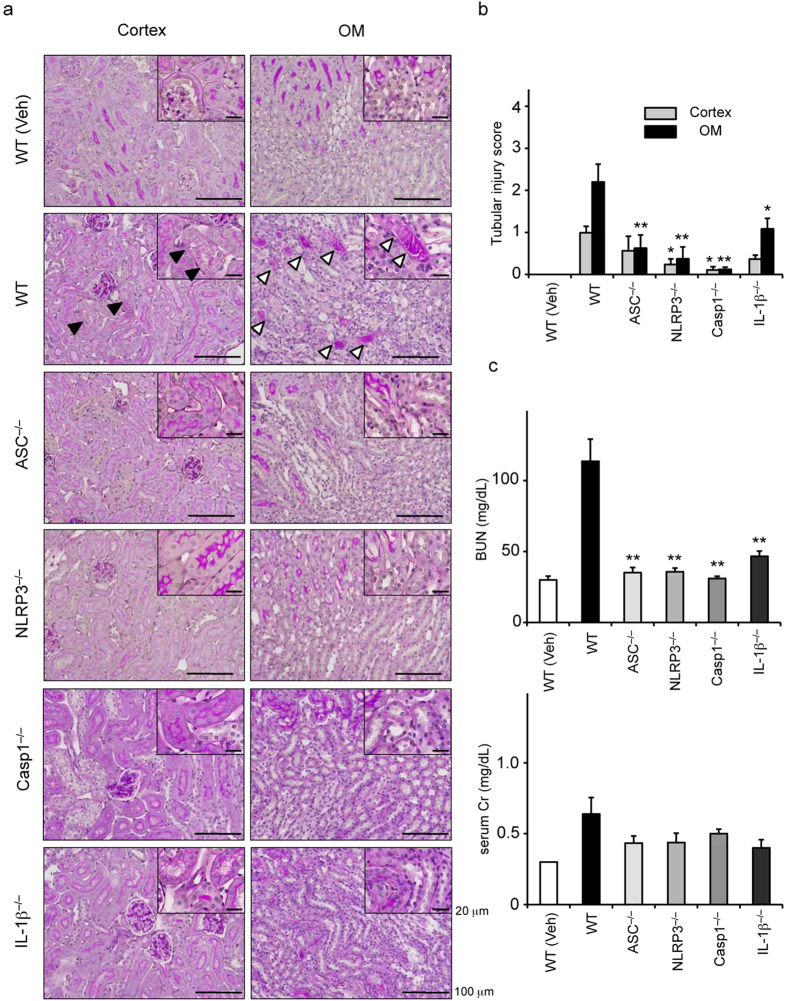
Effects of deficiency of NLRP3 inflammasome-related molecules on renal injury. Mice were sacrificed 24 h after 5 mL/kg glycerol (Gly) or vehicle (Veh) administration. The kidneys of wild-type (WT), ASC^–/–^, NLRP3^–/–^, Casp1^–/–^, and IL-1β^–/–^were obtained. (**a**) Representative images of PAS staining in the outer medulla are shown (black arrowheads, lytic lesions of the tubular epithelium; white arrowheads, intratubular casts). (**b**) The tubular injury score in the cortex and outer medulla (OM) was determined (n = 4–5). (**c**) Serum levels of BUN and Cr were assessed (n = 4, 13, 9, 8, 5, 4, respectively). Data are expressed as mean ± SEM and analyzed using ANOVA with Dunnett’s post-hoc test vs. WT. *p < 0.05, **p < 0.01.

**Figure 4:**
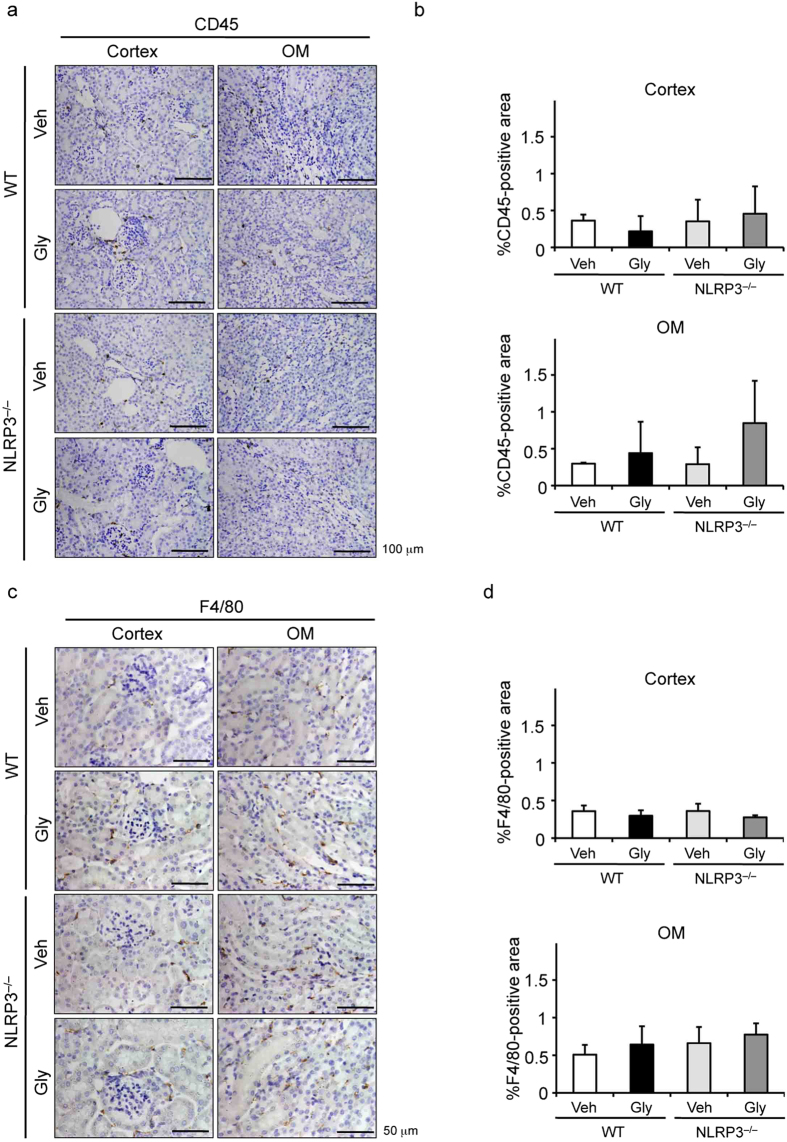
Effects of NLRP3 deficiency on leukocyte infiltration. Mice were sacrificed 24 h after administration of 5 mL/kg glycerol (Gly) or vehicle (Veh). The kidneys of WT and NLRP3^–/–^mice were obtained. (**a** and **c**) Representative photographs of immunostaining for CD45 (**a**) and F4/80 (**c**) in the cortex and outer medulla (OM) are shown. (**b** and **d**) A quantitative analysis of CD45 (**b**)- and F4/80 (**d**)-positive area in the cortex and outer medulla was performed (n = 4–5). Data are expressed as mean ± SEM.

To investigate the role of NLRP3 inflammasomes in the pathophysiology of RIAKI, we used mice deficient in NLRP3, ASC, caspase-1, and IL-1β, and generated RIAKI in these mice. Tubular injury determined by PAS staining was significantly attenuated in NLRP3^–/–^, ASC^−/–^, caspase-1^–/–^, and IL-1β^–/–^mice, compared with the injury in WT mice (Fig. 3a,b). Serum BUN levels were significantly lower in RIAKI of NLRP3^–/–^, ASC^–/–^, caspase-1^–/–^, and IL-1β^–/–^mice than that of WT mice (Fig. 3c). In addition, serum Cr levels were relatively but not significantly lower in these deficient mice (Fig. 3c). Because inflammatory cells play an important role in the development of RIAKI^21^, we measured the infiltration of leukocytes in the kidney of WT and NLRP3^–/–^mice. Immunohistochemical analysis for the pan-leukocyte marker CD45 and the macrophage marker F4/80 showed no significant increase of inflammatory cell infiltration in the cortex and outer medulla of both WT and NLRP3^–/–^mice at 24 h after RIAKI induction (Fig. 4). These results suggest that there is little contribution of infiltrated leukocytes to tubular injury during the early phase of RIAKI.

### Effects of NLRP3 deficiency on expression of tubular injury markers and inflammatory cytokines

**Figure 5:**
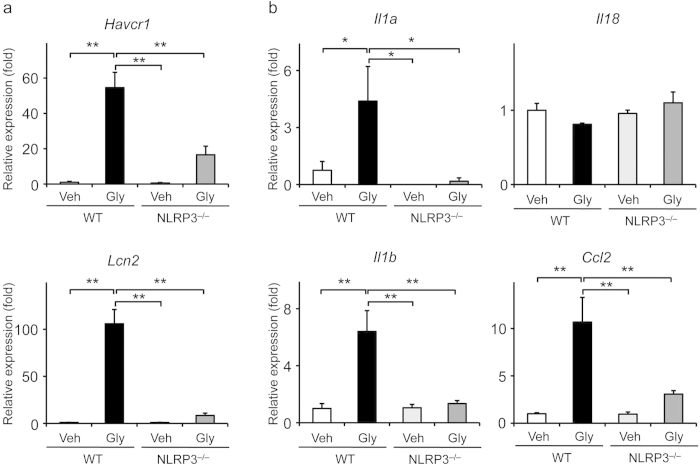
Effects of NLRP3 deficiency on expression of tubular injury markers and inflammatory cytokines. Mice were sacrificed 24 h after administration of 5 mL/kg glycerol (Gly) or vehicle (Veh). Renal mRNA levels of *Havcr1*, *Lcn2* (**a**), *Il1a*, *Il1b*, *Il18*, and *Ccl2* (**b**) were assessed by real-time RT-PCR analysis (n = 4–9). Data are expressed as mean ± SEM. *p < 0.05, **p < 0.01.

To investigate whether inflammatory responses are involved, we assessed the expression of tubular injury markers and inflammatory cytokines in the kidneys with RIAKI. Real-time RT-PCR analysis showed that the expression of *Havcr1* and *Lcn2* was markedly increased in the RIAKI kidneys of WT mice, but this increase was significantly inhibited in NLRP3^–/–^mice (Fig. 5a). The expression of *Il1a*, *Il1b*, *Il18*, and *Ccl2* was also significantly increased in WT mice; however, this expression was significantly inhibited in NLRP3^–/–^mice (Fig. 5B). Because NLRP3 inflammasomes regulate IL-1β release, we examined IL-1β expression in RIAKI kidney. Immunohistochemical analysis showed that IL-1β was expressed in distal tubules and cortical collecting ducts of the kidneys (Supplementary figure S2a). We also measured IL-1β levels in the renal tissue. Although no increase of renal IL-1β levels was observed during RIAKI, renal IL-1β levels were significantly less at 24 h in RIAKI kidneys compared with vehicle-treated kidneys (Supplementary figure S2b).

### Effects of NLRP3 deficiency on apoptosis

**Figure 6:**
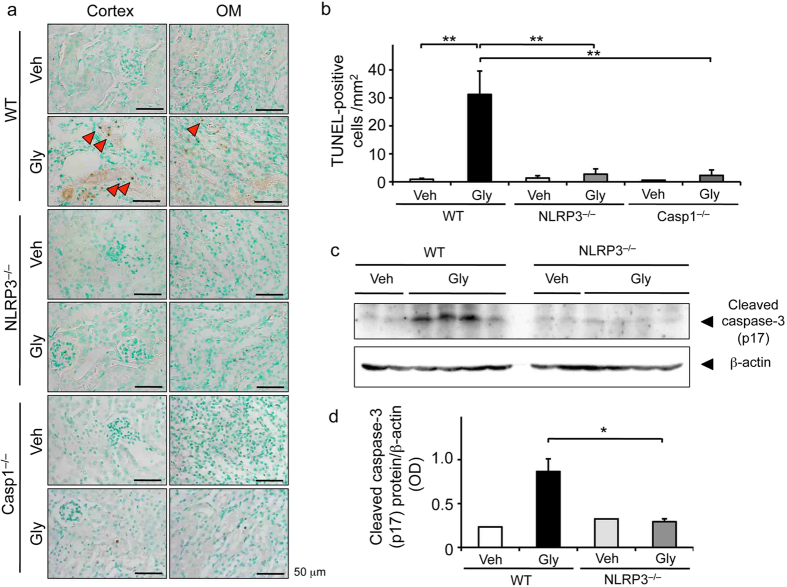
Effects of NLRP3 deficiency on apoptosis. Mice were sacrificed 24 h after 5 mL/kg glycerol (Gly) or vehicle (Veh) administration. The kidneys of wild-type (WT), NLRP3^–/–^, and caspase-1^–/–^ (Casp1^–/–^) were obtained. Paraffin-embedded sections of the kidney were stained with TUNEL staining to identify apoptotic cells. (**a**) Representative photographs of TUNEL staining. Arrowheads indicate TUNEL-positive cells. (**b**) Quantitative analysis of TUNEL-positive cells was performed (n = 4, 4, 4, 4, 2, 4, respectively). (**c**) Renal expression of cleaved caspase-3 was assessed by Western blot analysis. β-actin was used as the loading control. (**d**) Quantitative analysis of cleaved caspase-3 expression was performed (n = 2, 4, 2, 4, respectively). Data are expressed as mean ± SEM. *p < 0.05, **p < 0.01.

Since RIAKI has been shown to induce renal tubular apoptosis^22^,^23^, we assessed apoptosis by two protocols: a terminal transferase-mediated dUTP nick-end labeling (TUNEL) staining and western blotting for cleaved caspase-3, a marker for apoptosis. TUNEL staining showed that apoptotic cells were increased in cortical tubules of the RIAKI kidneys of WT mice, but the number of apoptotic cells was significantly lower than in that of NLRP3^–/–^ and caspase-1^–/–^ mice (Fig. 6a,b). Furthermore, the expression of cleaved caspase-3 was clearly detected in RIAKI kidneys of WT mice, whereas it was faint in the RIAKI kidney of NLRP3^–/–^mice (Fig. 6c,d, Supplementary figure S3).

To explore the cells expressing NLRP3, we finally performed immunohistochemical analysis for NLRP3. Serial sections that were stained for calbindin-D_28K_ (CaBP, a marker for distal convoluted tubule to connecting tubule) and aquaporin-2 (AQP2, a marker for collecting duct) indicate colocalization of NLRP3 with CaBP-D_28K_ and AQP2 (Supplementary figure S4). Consistent with previous reports^24^, NLRP3 was also detected in the glomeruli, suggesting the role of NLRP3 in renal resident cells.

## Discussion

Inflammation plays a pivotal role in the pathophysiology of RIAKI. In particular, recent studies have shown the importance of leukocytes, especially macrophages, in the development of RIAKI^21^. However, it is still unknown which mechanism is involved in the inflammatory responses associated with the development of RIAKI. In the present study, we demonstrated that renal inflammatory responses, tubular apoptosis, and tubular injury preceded apparent leukocyte infiltration into the kidney during the early phase of RIAKI and that these early phase manifestations were ameliorated in mice deficient in the NLRP3 inflammasome-related molecules, NLRP3, ASC, caspase-1, and IL-1β. To our knowledge, this study provides the first evidence that NLRP3 inflammasomes play a major role in the initial inflammation and injury of RIAKI. This study also suggests that renal tubular cells mediate the initial activation of NLRP3 inflammasomes. This is consistent with our recent report describing that ASC, a central component of NLRP3 inflammasomes, in renal collecting duct epithelial cells contributes to inflammation and injury after unilateral ureteral obstruction^17^. The results of the present study as well as our previous study provide new insights into the role of NLRP3 inflammasomes in kidney diseases.

Recent evidence indicates that NLRP3 inflammasomes play a pivotal role in the pathogenesis of renal disease^25^. In terms of AKI, NRLP3 inflammasomes have shown to mediate renal injury induced by I/R^26^ as well as folic acid treatment^27^. In the present study, we clearly demonstrated that deficiency of NLRP3, ASC, caspase-1, and IL-1β prevented the initial inflammatory responses and subsequent injury in RIAKI. Consistently, Homsi *et al.*^5^ reported that treatment with a caspase-1 inhibitor (Ac-YVAD-CHO) attenuates inflammatory responses and improves renal dysfunction in a rat model of RIAKI. Interestingly, however, Shigeoka *et al.*^28^ proposed that NLRP3 has a biological function independent of the NLRP3 inflammasomes because renal I/R injury was ameliorated in mice that were deficient in NLRP3, but not in ASC or caspase-1. In addition, Kim *et al.*^29^ reported that the NLRP3 inflammasomes make no contribution to the pathogenesis of nephrotoxic cisplatin-induced AKI. Therefore, we speculate that the contribution of NLRP3 inflammasomes may be influenced by differences in danger signals and the severity of experimental conditions in AKI models.

The NLRP3 inflammasomes are thought to play a role mainly in inflammatory cells^18^; however, recent investigations have suggested that initial inflammatory responses driven by NLRP3 inflammasomes can occur in tissue resident non-inflammatory cells. We previously reported that inflammasome activation of cardiac fibroblasts plays an essential role in the initial inflammatory responses and subsequent tissue damage after myocardial I/R injury^15^. Furthermore, we recently showed that inflammasome activation of renal collecting duct epithelial cells contributes to the initial inflammatory responses and renal injury after unilateral ureteral obstruction^17^. In the present study, we observed that deficiency of NLRP3, ASC, caspase-1, and IL-1β attenuated the initial inflammatory responses and injury in RIAKI before inflammatory cell infiltration, indicating a substantial role for renal resident cells (*e.g*., distal tubule and the collecting duct cells) during the early phase (<24 h) of RIAKI. However, we could not detect the NLRP3 inflammasome-dependent IL-1β processing and the apparent presence of mature IL-1β during RIAKI. In this regard, non-canonical effects of NLRP3 in tubular epithelial cells have recently received considerable attention^30^. Muruve’s group reported that NLRP3 enhances transforming growth factor-β (TGF-β) signaling in tubular epithelial cells and promotes renal fibrosis independently of the inflammasomes^31^,^32^. Similarly, Bakker *et al.*^33^ showed that tissue specific role of NLRP3 in tubular epithelial repair after renal I/R. Anders’s group also demonstrated that non-canonical effects of NLRP3 contribute to the pathogenesis of lupus nephritis and glomerulonephritis^34^,^35^. Furthermore, we have recently showed that NLRP3 regulates neutrophil functions and contribute to the pathogenesis of hepatic ischemia-reperfusion injury and hyperoxic acute lung injury independently of the inflammasomes^36^,^37^. Taken together, it is likely that NLRP3 has inflammasome-independent effects on renal inflammation and remodeling that are beyond the processing of IL-1β.

Renal tubular cell death is one of the prominent features in RIAKI^22^,^23^. We clearly showed that RIAKI-induced apoptotic cell death was almost completely inhibited by NLRP3 deficiency, indicating NLRP3 inflammasomes might regulate apoptosis in the kidney. Recently, another type of cell death, termed pyroptosis, is triggered by the inflammasome activation^38^. Pyroptosis is caspase-1-dependent and a highly inflammatory form of cell death, characterized by both apoptosis (*e.g.*, DNA fragmentation) and necrosis (*e.g.*, cell swelling and rupture). Therefore, TUNEL staining would not be able to distinguish between DNA fragmentation due to apoptosis or pyroptosis. To distinguish them, we assessed the active (cleaved) form of caspase-3 because its activation triggers apoptosis, but not pyroptosis; we then clearly showed an activation of caspase-3 in the RIAKI kidney. In addition, its activation was prevented by deficiency of NLRP3 and caspase-1, suggesting a role of NLRP3 and caspase-1 in the regulation of apoptosis. In this regard, Maedler *et al.*^39^ reported that high glucose resulted in the release of IL-1β, followed by NF-κB activation, Fas upregulation, and DNA fragmentation in islet cells of the pancreas. Therefore, we postulate that local IL-1β secretion driven by NLRP3 inflammasomes might induce apoptosis via a NF-κB/Fas-dependent pathway in the RIAKI kidney.

Several limitations of this study should be noted. First, the endogenous danger signals to activate NLRP3 inflammasomes during the early phase of RIAKI still need to be determined. In this regard, we examined whether a heme protein hemin, ferrous, or ferric myoglobin, all possible candidates that could be involved in RIAKI, could induce NLRP3 inflammasome activation in primary tubular and collecting duct epithelial cells *in vitro*. However, these stimuli did not induce its activation (data not shown). In addition, although IL-1β release does need a two-step process requiring a priming step (signal 1) to synthesize pro-IL-1β, followed by an inflammasome-mediated processing step (signal 2), the priming stimuli have not been currently unidentified. Second, although upregulation of IL-1β mRNA expression was detected in the kidneys with RIAKI, the processing of pro-IL-1β as well as the elevation of the IL-1β at protein levels could not be confirmed *in vivo* due to undetectable levels (data not shown). Third, because we could not detect IL-1β protein release during RIAKI, we could not rule out the non-canonical effects of NLRP3 at the early phase of RIAKI, as described above. Thus, further studies will be necessary to elucidate the precise role of NLRP3 inflammasomes in the pathogenesis of RIAKI.

In conclusion, we showed that NLRP3 inflammasomes initiate inflammatory responses and apoptotic cell death, resulting in tubular injury during the early phases of RIAKI. This initial activation of NLRP3 inflammasomes might be involved in renal tubular cells, and stimulate the release of cytokines/chemokines; this in turn can recruit inflammatory cells, such as monocytes/macrophages, and enhance inflammatory responses, and subsequent renal injury. The present results not only suggest NLRP3 inflammasomes may be a potential therapeutic target for prevention and treatment of RIAKI but may also provide new insights into the mechanism underlying the pathogenesis of RIAKI.

## Methods

### Animals and RIAKI model

All animal experiments were approved by the Use and Care of Experimental Animals Committee of the Jichi Medical University Guide for Laboratory Animals, and were carried out in accordance with the Jichi Medical University guidelines. ASC^–/–^mice were generated as described previously^40^. NLRP3^–/–^, caspase-1^–/–^, and IL-1β^–/–^mice were kindly provided by Dr. Vishava M. Dixit (Genentech, South San Francisco, CA), Dr. H. Tsutsui (Hyogo College of Medicine, Nishinomiya, Japan), and Dr. Yoichiro Iwakura (Tokyo University of Science, Chiba, Japan), respectively^41^,^42^,^43^,^44^. C57BL/6J wild-type (WT) mice were purchased from Japan SLC, Inc. (Tokyo, Japan). Mice were fed, watered, and maintained on a 12-h light and dark cycle. RIAKI was induced by intramuscular administration of 50% glycerol in 8–10 week-old male mice (C57BL/6J genetic background) as previously described^45^. Briefly, mice were deprived of water 24 h before the glycerol administration. Glycerol (50%, 5 mL/kg) or vehicle (0.9% saline) was intramuscularly administered as equally divided doses into the hind limbs.

### Renal functions

Serum levels of BUN and Cr were measured by using the chemical analyzer Fuji Dry-Chem (Fujifilm, Tokyo, Japan) according to the manufacturer’s instructions.

### Real-time RT-PCR analysis

Total RNA was prepared from the kidney using ISOGEN (Nippon Gene Co., Ltd., Toyama, Japan) according to the manufacturer’s instructions. Real-time RT-PCR analysis was performed using the Takara TP960 PCR Thermal Cycler Dice Detection System (Takara Bio Inc, Shiga, Japan) to detect mRNA expression. The following primers (oligonucleotide sequences are provided in parentheses in the order of sense and antisense primers) were used: *Nlrp3* (5′-CGAGACCTCTGGGAAAAAGCT-3′ and 5′-GCATACCATAGAGGAATGTGATGTACA-3′), *Asc* (5′-GCTGAGCAGCTGCAAACGAC-3′ and 5′-ACTTCTGTGACCCTGGCAATGAG-3′), *Casp1* (5′-GATGGCACATTTCCAGGACTGA -3′ and 5′-TGTTGCAGATAATGAGGGCAAGAC -3′), *Il1b* (5′-TGAAGTTGACGGACCCCAAA-3′ and 5′-TGATGTGCTGCTGTGAGATT-3′), *Havcr1* (5′-CTGGAATGGCACTGTGACATCC-3′ and 5′-GCAGATGCCAACATAGAAGCCC-3′), *Lcn2* (5′-GAAATATGCACAGGTATCCTC-3′ and 5′-GTAATTTTGAAGTATTGCTTGTTT-3′), *Il1a* (5′-AGCGCTCAAGGAGAAGACC-3′ and 5′-CCAGAAGAAAATGAGGTCGG -3′), *Il18* (5′-CAGGCCTGACATCTTCTGCAA-3′ and 5′-TCTGACATGGCAGCCATTGT-3′), *Ccl2* (5′-GGCTCAGCCAGATGCAGTTAAC-3′ and 5′-GCCTACTCATTGGGATCATCTTG-3′), and *Gapdh* (5′-TGTGTCCGTCGTGGATCTGA-3′ and 5′-TTCGTGTTGAAGTCGCAGGAG-3′). The expression levels of each target gene were normalized by subtracting the corresponding glyceraldehyde-3-phosphate dehydrogenase (GAPDH) threshold cycle (C_T_) values; this was done by using the ∆∆C_T_ comparative method. WT vehicle control was used to calculate relative changes in PCR data.

### Histology and immunohistochemistry

After the mice were euthanized and perfused with 0.9% saline, the kidneys were harvested, fixed in 10% formalin, and embedded in paraffin. The sections (2-μm thick) were used for staining with PAS using a standard protocol. At least 5 images were randomly captured with 200× magnification using the Leica Application Suite software (ver.3.4.1, Leica Microsystems GmbH, Wetzlar, Germany). Tubular injury was indicated on PAS samples by necrotic lysis, tubular dilatation, tubular brush border loss, cast formation, and sloughing of cellular debris into the tubular lumen. The scoring was performed as described previously^46^ according to the following grades: normal as grade 0; <25% as grade 1; 25%–49% as grade 2; 50%–74% as grade 3; and ≥75% as grade 4.

Immunohistochemistry of the paraffin-embedded sections were performed using a polymeric method. The deparaffinized kidney sections were boiled in Target Retrieval Solution (Dako, Carpinteria, CA), blocked with a 5% normal serum of the secondary antibody species, and incubated overnight in the primary antibodies. This was followed by incubation with Histofine Simple Stain MAX PO (Nichirei Bioscience, Tokyo, Japan). The reaction was developed using a DAB substrate kit (Vector Laboratories), followed by hematoxylin counterstaining. No signals were detected when species-matched immunoglobulin G (IgG) (Vector Laboratories) was used instead of the primary antibody as a negative control. Primary antibodies included the following: rat anti-mouse CD45 antibody (BD Biosciences, San Jose, CA), monoclonal rat anti-mouse F4/80 antibody (clone A3-1; AbD Serotec), polyclonal rabbit anti-mouse IL-1β antibody (Santa Cruz Biotechnology, TX), goat anti-CIAS1/NALP3 antibody (Abcam, Cambridge, UK), polyclonal goat anti-aquaporin-2 antibody (AQP2 [C-17]; Santa Cruz Biotechnology, TX), and polyclonal goat anti-calbindin D_28K_ antibody (CaBP [C-20]; Santa Cruz Biotechnology). At least 10 random fields of 200× magnification were analyzed for measurement in a similar manner. The stained sections were digitalized and analyzed using a microscope (FSX-100, Olympus, Tokyo, Japan). The CD45-positive area, F4/80-positive area, and cleaved caspase-3-positive area were measured using ImageJ 1.47v (U.S. National Institute of Health, Bethesda, MD).

### Western blot analysis

The expression of ASC, NLRP3, and cleaved caspase-3 was analyzed by Western blotting. Protein lysates were prepared from the kidney using RIPA buffer (20 mM Tris, 2.5 mM EDTA, 1% Triton X, 10% glycerol, 1% deoxycholic acid, 0.1% SDS, 50 mM NaF, and 10 mM Na_4_P_2_O_7_·10H_2_O) and subjected to sodium dodecyl sulfate polyacrylamide gel electrophoresis (SDS-PAGE); the protein bands were transferred to polyvinylidene difluoride (PVDF) membranes. The membranes were blocked overnight at 4 °C with Blocking One solution (Nakarai Tasque, Shiga, Japan) and then incubated for 1 h at room temperature with the primary antibodies, followed by incubation for 1 h with the secondary antibody, conjugated horseradish peroxidase (HRP). Primary antibodies against ASC (Enzo Life Sciences, Inc.), NLRP3 (R&D Systems), cleaved caspase-3 (Asp-175, Cell Signaling Technologies, Denver, MA), and β-actin (Sigma) were used. The secondary antibody was HRP-conjugated anti-IgG (Jackson ImmunoResearch Laboratories, Inc., PA). The immunoreactive bands were visualized by a Western BLoT HRP Chemiluminescent Substrate system (Takara Bio, Shiga, Japan). The expression levels of β-actin served as an internal control for protein loading.

### Detection of apoptosis

Apoptotic cells were identified with TUNEL staining. An *in situ* Apoptosis Detection kit (Takara Bio, Tokyo, Japan) was used according to the manufacturer’s instructions.

### Statistics

Data were expressed as mean ± standard error of the mean (SEM). An unpaired *t* test was used to compare two groups. For comparisons between multiple groups, the significance of differences in between-group means was determined by one-way analysis of variance (ANOVA) combined with the Turkey-Kramer test or Dunnett post-hoc test. Homogeneity of variance was assessed by the F-test or the Brown-Fortsythe test. Nonparametric data were analyzed using the Mann-Whitney U test or the Kruskal-Wallis test as appropriate. All analyses were performed using the Graphpad Prism version 6 (San Diego, CA). A *p*-value of *< *0.05 was considered statistically significant.

## Additional Information

**How to cite this article**: Komada, T. *et al.* Role of NLRP3 Inflammasomes for Rhabdomyolysis-induced Acute Kidney Injury. *Sci. Rep.* **5**, 10901; doi: 10.1038/srep10901 (2015).

## Supplementary Material

Supplementary Information
