# Supplementary material for: Role of NLRP3 Inflammasomes for Rhabdomyolysis-induced Acute Kidney Injury
Source: Sci Rep. 2015 Jun 5;5:10901. doi: 10.1038/srep10901 (PMC4456665; doi:10.1038/srep10901)
Supplement: Supplementary Information [file srep10901-s1.pdf]

**Role of NLRP3 Inflammasomes for Rhabdomyolysis-induced Acute Kidney Injury**

Takanori Komada[1,2*], Fumitake Usui[1*], Akira Kawashima[1], Hiroaki Kimura[1], Tadayoshi Karasaw [1], Yoshiyuki Inoue[1], Motoi Kobayashi[1], Yoshiko Mizushina[1], Tadashi Kasahara[1], Shun'ichiro Taniguchi[3], Shigeaki Muto[2], Daisuke Nagata[2], Masafumi Takahashi[1]

[1]Division of Inflammation Research, Center for Molecular Medicine,
[2]Department of Nephrology, Jichi Medical University, Tochigi;
[3]Department of Molecular Oncology, Shinshu University Graduate School of Medicine, Nagano; Japan
*These authors contributed equally.

Correspondence: Masafumi Takahashi, MD, PhD, Division of Inflammation Research, Center for Molecular Medicine, Jichi Medical University
3311-1 Yakushiji, Shimotsuke, Tochigi 329-0498, Japan
E-mail: masafumi2@jichi.ac.jp

## Supplementary information

*Supplementary figure S1. NLRP3 protein expression in vehicle-treated kidney*

The kidneys were obtained from WT mice at the indicated time points after glycerol (Gly) or vehicle (Veh) administration. Protein samples were prepared from the kidneys. NLRP3 and ASC expressions were assessed by Western blot analysis. β-actin was used as the loading control. Representative images are shown.

*Supplementary figure S2. IL-1β levels in glycerol- or vehicle-treated kidneys*

The kidneys were obtained from WT mice at the indicated time points after glycerol (Gly) or vehicle

(Veh) administration. (a) Expression of IL-1β was assessed by imunostaining. (a) Representative images are shown. (b) Protein samples were prepared from the kidneys. Renal IL-1β levels were assessed (n = 3–4). Data are expressed as mean ± SEM. ***p* < 0.01 vs. Veh.

*Supplementary figure S3. Immunohistochemistry for cleaved aspase-3*

The kidneys were obtained from WT, NLRP3$^{-/-}$, and Casp1$^{-/-}$ mice 24 h after glycerol (Gly) or vehicle (Veh) administration. Expression of cleaved caspase-3 was assessed by immunostaining. (a) Representative images are shown. (b) Quantitative analysis of cleaved caspase-3-positive area was performed (n = 6, 5, 4, 5, 1, 4, respectively). Data are expressed as mean ± SEM. **p* < 0.05, ***p* < 0.01.

*Supplementary figure S4. Immunohistochemistry for NLRP3*

The kidneys were obtained from WT mice 24 h after 5 mL/kg glycerol (Gly) or vehicle (Veh) administration. (a) The sections were immunohistochemically stained with an antibody against NLRP3. Representative photographs are shown (green arrowheads, intraglomerular NLRP3-positive cells; red arrowheads, NLRP3 positive tubules). (b) The serial sections were stained for NLRP3, CaBP-$D_{28K}$ (a marker for distal convoluted tubule and connecting tubule), and AQP2 (a marker for collecting duct). Isotype IgG was used for control staining. Representative photographs are shown.

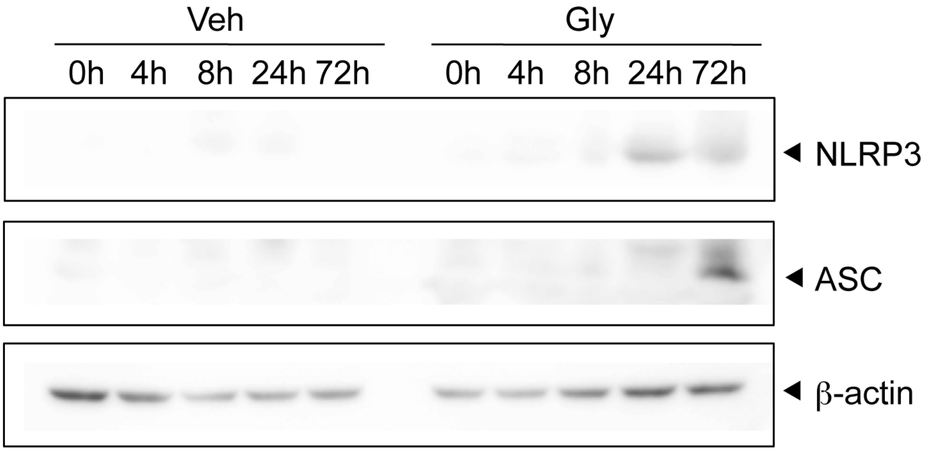
Veh
Gly
0h 4h 8h 24h 72h
0h 4h 8h 24h 72h
NLRP3
ASC
β-actin

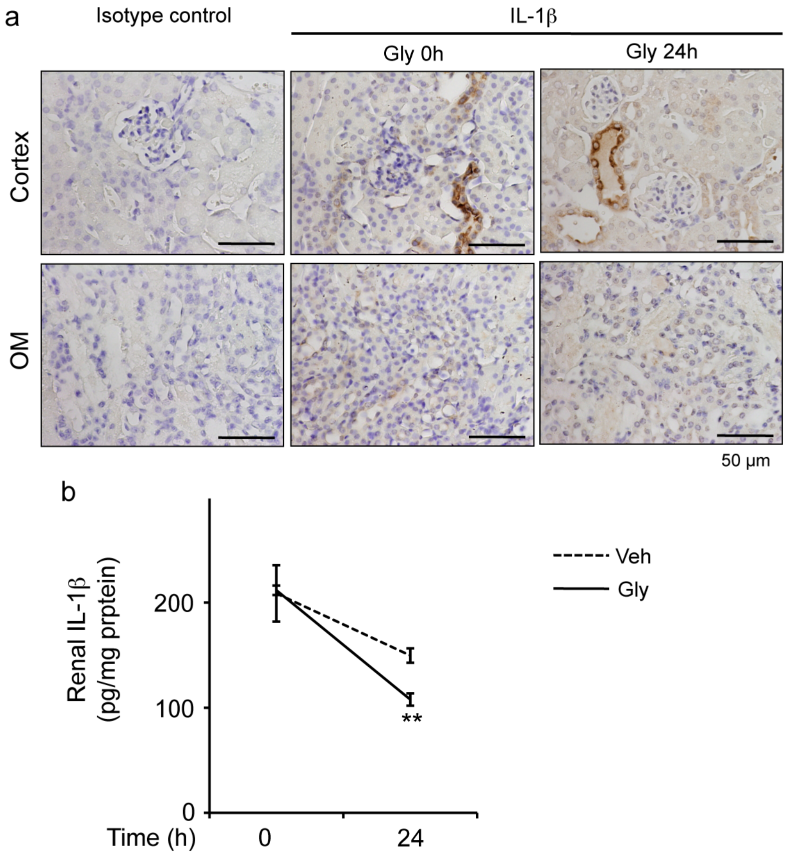
a
Isotype control
IL-1β
Gly 0h
Gly 24h
Cortex
OM
50 μm
b
Renal IL-1β
(pg/mg prptein)
200
100
0
Time (h)
0
24
**
Veh
Gly

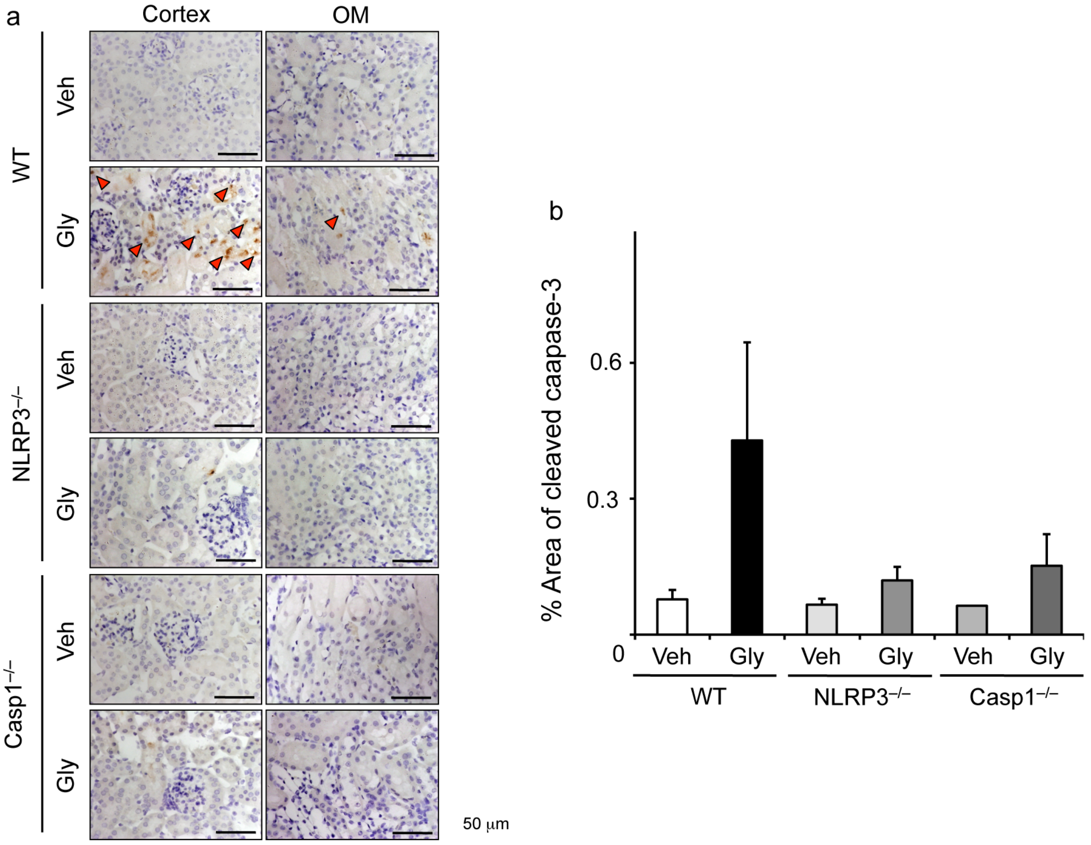
a
Cortex
OM
WT
Veh
Gly
NLRP3−/−
Veh
Gly
Casp1−/−
Veh
Gly
50 μm
b
% Area of cleaved caapase-3
0.6
0.3
0
Veh
Gly
Veh
Gly
Veh
Gly
WT
NLRP3−/−
Casp1−/−

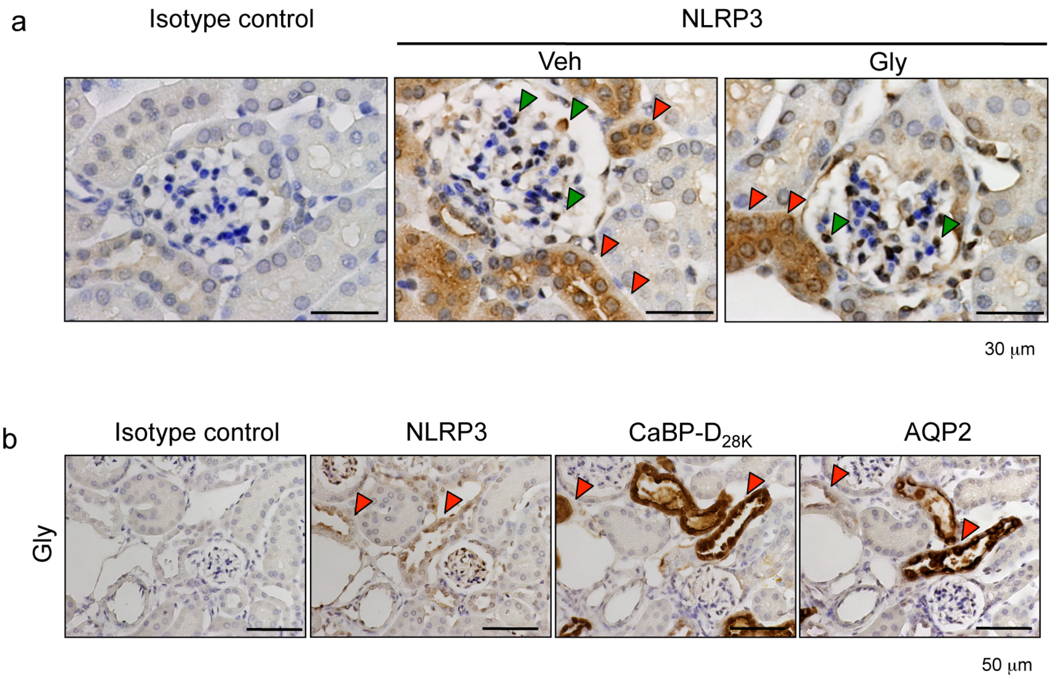
a
Isotype control
NLRP3
Veh
Gly
30 μm
b
Isotype control
NLRP3
CaBP-D28K
AQP2
Gly
50 μm
